# Feedforward Inhibition and Synaptic Scaling – Two Sides of the Same Coin?

**DOI:** 10.1371/journal.pcbi.1002432

**Published:** 2012-03-22

**Authors:** Christian Keck, Cristina Savin, Jörg Lücke

**Affiliations:** 1 Frankfurt Institute for Advanced Studies, Frankfurt am Main, Germany; 2 Computational and Biological Learning Lab, Department of Engineering, University of Cambridge, Cambridge, United Kingdom; 3 Department of Physics, Goethe-University, Frankfurt am Main, Germany; Indiana University, United States of America

## Abstract

Feedforward inhibition and synaptic scaling are important adaptive processes that control the total input a neuron can receive from its afferents. While often studied in isolation, the two have been reported to co-occur in various brain regions. The functional implications of their interactions remain unclear, however. Based on a probabilistic modeling approach, we show here that fast feedforward inhibition and synaptic scaling interact synergistically during unsupervised learning. In technical terms, we model the input to a neural circuit using a normalized mixture model with Poisson noise. We demonstrate analytically and numerically that, in the presence of lateral inhibition introducing competition between different neurons, Hebbian plasticity and synaptic scaling approximate the optimal maximum likelihood solutions for this model. Our results suggest that, beyond its conventional use as a mechanism to remove undesired pattern variations, input normalization can make typical neural interaction and learning rules optimal on the stimulus subspace defined through feedforward inhibition. Furthermore, learning within this subspace is more efficient in practice, as it helps avoid locally optimal solutions. Our results suggest a close connection between feedforward inhibition and synaptic scaling which may have important functional implications for general cortical processing.

## Author Summary

The inputs a neuron receives from its presynaptic partners strongly fluctuate as a result of either varying sensory information or ongoing intrinsic activity. To represent this wide range of signals effectively, neurons use various mechanisms that regulate the total input they receive. On the one hand, feedforward inhibition adjusts the relative contribution of individual inputs inversely proportional to the total number of active afferents, implementing a form of input normalization. On the other hand, synaptic scaling uniformly rescales the efficacy of incoming synapses to stabilize the neuron's firing rate after learning-induced changes in drive. Given that these mechanisms often act on the same neurons, we ask here if there are any benefits in combining the two. We show that the interaction between the two has important computational consequences, beyond their traditional role in maintaining network homeostasis. When combined with lateral inhibition, synaptic scaling and fast feedforward inhibition allow the circuit to learn efficiently from noisy, ambiguous inputs. For inputs not normalized by feed-forward inhibition, learning is less efficient. Given that feed-forward inhibition and synaptic scaling have been reported in various systems, our results suggest that they could generally facilitate learning in neural circuits. More broadly, our work emphasizes the importance of studying the interaction between different plasticity mechanisms for understanding circuit function.

## Introduction

As part of an ever-changing world, brain activity changes continuously. The fraction of neurons active in a region at each given moment fluctuates significantly driven by changes in the environment and intrinsic dynamics. Ideally, regions receiving this activity as input should be able to represent incoming signals reliably across the full possible range of stimulation conditions. Indeed, this type of regulation seems to be ubiquitous in the cortex. In the early visual system, contrast gain control begins in the retina [1] and is strengthened at subsequent stages of the visual system, such that the way an image is represented in V1 simple cells is largely contrast invariant [2], [3]. Similarly, in the olfactory system, neuronal representations remain sparse and odor-specific over thousand-fold changes in odor concentration [4]–[6].

To be able to achieve such invariance, neurons have evolved various mechanisms that adjust neuronal response properties as function of their total input. One instance of such normalization involves feedforward inhibition, in which afferent inputs induce both excitation and mono-synaptically delayed inhibition onto principal cells [7]–[12], shaping the temporal activity pattern of the postsynaptic neurons [8]–[10], and sparsifying population activity [5]. The degree of specificity of this inhibition can vary from stimulus specific to relatively unspecific [7], [12]. Here, we focus on fast but unselective feedforward inhibition, which has been reported in a range of circuits including hippocampus and sensory areas [11], [13]–[15]. This mechanism adjusts, virtually instantaneously, the sensitivity of pyramidal cells to the overall strength of the afferent stimulus. As a result, the influence of an individual afferent on the firing of the postsynaptic neuron is continuously normalized by the total number of active afferents. Functionally, it has been hypothesized that such input normalization is needed to expand the range of inputs that can be represented in a neuron population [11], however, its implications for learning in the circuit remain unclear.

Another mechanism with similar effects, but acting on a slower time scale, is synaptic scaling [16]–[18]. Specifically, it is believed that neurons detect sustained changes in their firing rates through calcium-dependent sensors and increase or decrease the density of glutamate receptors at synaptic sites to compensate for these changes in drive [19]. This results in an uniform rescaling of the strength of excitatory synapses as a function of average postsynaptic activity. Synaptic scaling often takes a multiplicatively form [17], which has the benefit of preserving the relative contribution of synapses and hence the information stored through Hebbian learning [20]. This type of weight normalization is believed to address a different kind of stability problem–the fact that synapses are plastic. As Hebbian learning alone would destabilize neural dynamics, due to a positive feedback loop, additional homeostatic mechanisms such as synaptic scaling are needed to ensure stable circuit function [18]–[20].

Fast feedforward inhibition and synaptic scaling have been reported for a range of circuits including hippocampal and neocortical pyramidal neurons [11], [19]. Given that both mechanisms effectively regulate the total incoming drive to neurons, it may be somewhat surprising that they co-occur in the same cell types. This suggests there may be some computational advantage in combining input normalization and synaptic scaling. However, based on the existing experimental evidence alone, it is unclear what possible benefits this interaction may have.

We show here that the role of input normalization and synaptic scaling goes beyond simply maintaining circuit homeostasis, and that they play important computational roles during synaptic learning. In the presence of neuronal competition by global lateral inhibition, the two enable efficient unsupervised learning from noisy or ambiguous inputs. Specifically, we consider an elementary circuit that incorporates synaptic scaling and fast feedforward inhibition. We analyze the learning dynamics in this circuit and show that, for certain input statistics, standard neural dynamics and Hebbian synaptic plasticity implement approximately optimal learning for this data–an observation that we further confirm in numerical experiments. The studied circuit learns an efficient representation of its inputs which can be used for further processing by downstream networks (e.g., for classification). Importantly, in the absence of feedforward inhibition, learning in the same circuit results in much poorer representations, as the system has a stronger tendency to converge to locally optimal solutions–a problem that neural and non-neural systems for unsupervised learning commonly face. This provides evidence for synaptic plasticity requiring normalized inputs for efficient learning. Given that feedforward inhibition and synaptic scaling seem to co-occur in various neural circuits, our results suggest that the interplay between the two mechanisms may generally facilitate learning in the cortex.

## Results

We construct a model of feedforward inhibition and synaptic scaling acting in a neural circuit in which excitatory synapses change by Hebbian learning. The analysis of their interaction proceeds in two steps. First, we study the dynamics of learning within the circuit, leaving details of the neural dynamics unspecified. This analysis reveals that the weights converge to final values that are fully determined by the input distribution and the neuronal transfer function. Second, when using a specific statistical model for the input distribution, we can identify biologically plausible neural dynamics that implement optimal learning for these stimuli. We show that a specific form of lateral inhibition implementing softmax competition between different neurons is sufficient for optimal learning in our setup, something which we then confirm by numerical simulations, using both artificially generated and natural data. Lastly, we show that learning performance is critically dependent on feedforward inhibition and, how the emerging representations can be used by higher processing layers, for instance, for efficient classification.

### A neural circuit model

**Figure 1:**
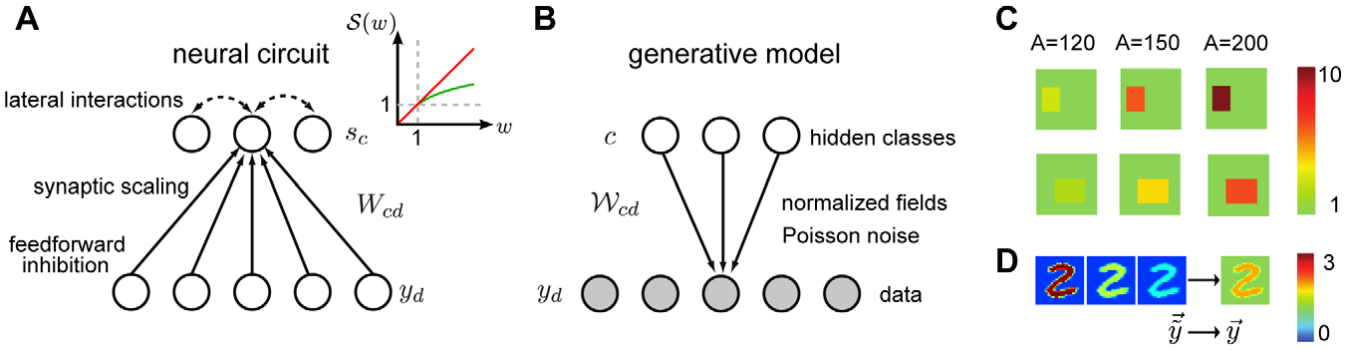
An overview of the model. (A) The neural circuit receives normalized inputs conveyed by excitatory synapses to a processing layer (large figure). The activity of the processing neurons is determined by the received inputs and internal dynamics mediated by lateral interactions. Inset: Two forms of weight scaling. The red curve shows conventional linear scaling, the green curve logarithmic scaling for values larger one. (B) Inputs to the circuit are modeled using a mixture model with normalized generative fields and Poisson noise. (C) Example normalized fields, with different values of the normalization constant 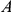. (D) Illustration how inputs with different contrast levels are normalized (background set to 1).

As a starting point, consider the elementary neural circuit shown in Fig. 1A. The network consists of 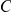 neurons receiving excitatory inputs from 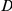 input neurons through a set of excitatory weights 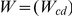, 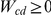. We denote by 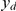 the activity of input neuron 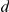 and by 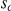 the activity of the downstream processing neuron 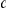.

In the general case, the activity of neurons 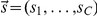 can be defined as a function of the activity of the input layer, 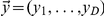, and of the weights 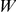:
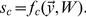
(1)This transfer function is not necessarily local, as it does not restrict the dependency to the afferent weights of neuron 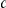; it allows us to also describe the interactions between neurons through lateral connections (marked by dotted lines in Fig. 1A). For the first part of the analysis, we assume the neural dynamics given by (1) to be arbitrary, though later we consider specific forms for the transfer function.

We model feedforward inhibition by explicitly normalizing the input vector 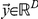 to satisfy the constraint:
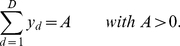
(2)Such input normalization can remove undesired patterned variations (e.g. contrast, see Fig. 1D), potentially facilitating learning in the circuit. If we denote the un-normalized input by 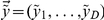, the constraint can, for instance, be fulfilled by a simple division, 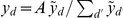, though alternative implementations are possible. This formulation abstracts away the details of the biological implementation, focusing on its functional implications [11]. Importantly, the simple form allows us to derive theoretical results about the role of this form of feedforward inhibition during learning. At the level of the neural circuit, however, input normalization relies on the presence of a set of fast spiking interneurons (in the hippocampus – predominantly basket cells [8]) innervated by the same afferent inputs, with unspecific projections onto the subsequent layer. The implications of this neural implementation are considered in more detail in the Discussion.

We model incoming synapses to be plastic and to change by Hebbian learning, with synaptic scaling implemented by an additional weight dependent term [20], [21]:
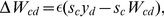
(3)where 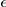 is a small positive learning rate. This synaptic scaling model captures the important biological constraint that weight changes should rely only on information that is local to the synapse. It differs from global forms that use an explicit weight normalization in that the normalizing constant is not a separate parameter, but rather is implicitly determined by the circuit dynamics.

### Evolution of weights during learning

The circuit model above defines specific learning dynamics for the synaptic weights as function of the their initial values and of the incoming inputs 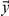. To investigate the evolution of the weights analytically, it is informative to first study the time course of the weight sums 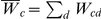 for an arbitrary neuron 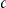. Using the learning rule (Eq. 3) and the explicit input normalization constraint (Eq. 2), we obtain:
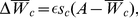
(4)which shows that 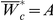 is a stationary point for the dynamics of 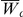. Furthermore, since neural activity and the learning rate are both positive, 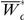 is a stable stationary point, i.e., 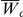 increases when smaller than 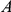, and decreases when larger, independent of the input statistics. Consequently, synaptic plasticity automatically adjusts the sum of the incoming weights to each neuron to the total incoming drive (since 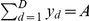). Hence, the synaptic weights of a processing neuron adapt during learning to match the scale of its inputs. Rather than being a separate parameter, the norm of the weights is inherited from the properties of the input stimuli. We show below that this match of the normalizing constants for inputs and weights, respectively, is critical for achieving efficient learning in the neural circuit.

In contrast to the mean 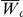, which is independent of the inputs 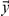 provided that the inputs are normalized, the stationary points for individual weights 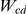 depend on the statistics of the input patterns. Such a dependency is, of course, needed if the circuit is to memorize properties of the input after learning. We can derive an analytical solution for learning in this system, something that has often proved difficult for other models. Specifically, if we consider the input vectors 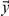 to be drawn independently and identically from a stationary but otherwise unspecified distribution 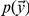, we can show (see Methods) that, at convergence, the weights associated with each neuron are uniquely determined by the statistics of input stimuli and the transfer function 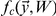:
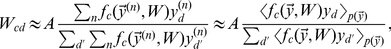
(5)where the brackets denote the average of the expression under the input distribution. This approximation is very accurate for small learning rates 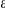 and large numbers of inputs.

### A statistical model for normalized input stimuli

Although Eq. 5 gives a formal description for the outcome of learning in the neural circuit as a function of the neuron dynamics 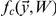 and the input statistics 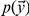, it tells us little about the quality of the learning result. For this, we need to specify the input distribution 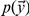. In particular, we use a generative model, which gives not only an explicit model for the input statistics 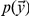, but also an expression for the theoretically optimal solution for inference and learning on such data, which we can use to evaluate the quality of learning in the neural circuit [22].

The specific generative model we chose is a mixture model, which is naturally associated with classification tasks [23]. Intuitively, a mixture model assumes each input stimulus to belong to one out of 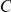 classes. Each class is described by a representative input and its typical variations. Mixture models have been well-investigated theoretically and are used to model a variety of data [23]. Moreover, although they may seem restrictive, mixtures are well-suited to model multi-modal data distributions also when the assumptions of the model are not satisfied exactly [23].

In generative model terminology, mixture distributions assume an input 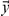 to be generated by one of 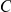 model classes (see Fig. 1B). Each class 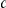 is described by a representative pattern 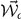, which we will refer to as its generative field. The mixture distributions 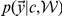 define the variations of the patterns within each class, where 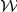 is the matrix of all generative fields. The prior probability 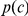 specifies how many inputs are generated by the different classes. Here, we assume all classes to be equally likely, and, since inputs 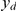 represent positive firing rates, we choose the Poisson distribution to model noise:
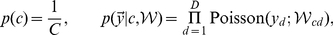
(6)where 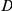 is the number of input dimensions.

To capture the effects of feedforward inhibition, we assume the parameters 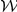 to satisfy the constraint:
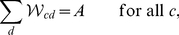
(7)with parameter 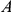 effectively determining the contrast of the inputs, see Fig. 1C. Note that this model only approximates the effect of feedforward inhibition, since individual stimuli are not normalized (the constraint in Eq. 2 is only true on average). However, the approximation gets increasingly accurate with increasing size of the stimuli, 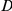.

Having a model for the input distribution, we can derive the optimal solution for inference and learning on this data. In particular, we use the expectation maximization (EM) framework [24], [25] which enables us to learn the maximum likelihood solutions for the parameters 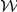 from input stimuli. Intuitively, this optimal learning procedure alternates between what we call the E-step, estimating how likely the data are under the current model, and the M-step, when we change the model parameters. Iterating E- and M-steps is guaranteed to never decrease the data likelihood and, in practice, it increases the likelihood to (possibly local) likelihood maxima. If a global maximum likelihood solution is found, the parameters 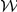 represent the best possible learning result (in the limit of many data points). Similarly, the posterior distribution with optimal 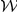 represents the best possible inference given any specific input. For our model, we obtain the following update rules for optimal parameter learning:
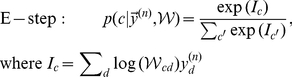
(8)

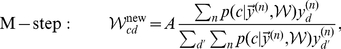
(9)where the posterior probability required for the E-step takes the form of the well-know softmax function [26] with arguments 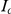.

### Optimal learning in the neural circuit

With the concrete model of normalized input data, we can now ask how learning in our neural circuit is related to the theoretically optimal solutions for such data. First, recall that after learning in the neural circuit has converged, the synaptic weights are a solution of Eq. 5. Second, for the probabilistic model the (possibly local) optimum is obtained after the EM iterations have converged, which means that 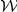 satisfies Eq. 9 with 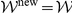. Comparing the result of neural learning with the result of EM learning, we note that they have a very similar structure:
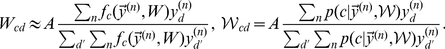
(10)Indeed, synaptic weights 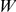 can be easily mapped into the parameters 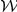 of the generative model and if we choose the transfer function 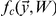 in the circuit to be equal to the posterior probability 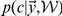, the two expressions are the same. Hence, if we interpret neural activity as representing posterior probabilities under our model (compare [27]–[31]), any fixed point of EM optimization becomes an approximate fixed point of neural learning.

The transfer function 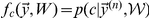 makes learning in the neural circuit approximately optimal for normalized data, but what does this transfer function mean in neural terms? First, the optimal neural dynamics requires a specific form of lateral interactions, implementing the softmax function (Eq. 8, left-hand-side). Through these interactions, neurons compete for representing each input stimulus. Due to its importance for competitive learning, neural circuits giving rise to the softmax have extensively been investigated [26], [32]–[34]. Typically they involve unspecific feedback inhibition which suppresses neurons with weak inputs while those with strong inputs can maintain high activity rates. Most of the variants of the implementation should work for the purposes of our model (also compare [35]–[37]); hence we do not commit to one specific realization of this function.

The arguments of the softmax have a particularly simple form: they represent local summations of input activities weighted by synaptic strengths, 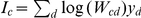. While the summation of inputs is biologically plausible, scaling by the logarithm of the weights 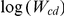 may not be. It, for instance, implies that the contribution of an input to a neuron's activity may be negative or, unrealistically, change sign during learning. This problem can be addressed, however, while preserving the close correspondence between the circuit's fixed points and maximum likelihood solutions. To achieve this, we note that the only requirement for the input data 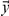 is that the total input is preserved, 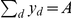. We therefore have some freedom when modeling how feedforward inhibition enforces this constraint. In particular, if the un-normalized input is 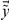, then feedforward inhibition could constrain the total inputs by:
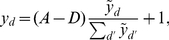
(11)which represents a slight alteration to the common choice 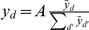. Practically, this form of normalization continues to scale the activity of an un-normalized input unit 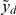 by the total activity 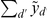, but it introduces an offset corresponding to having some spontaneous background activity in the input layer (which leads to a normalization constant 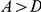).

This model of feedforward inhibition guarantees that the weights will eventually converge to values larger or approximately equal to one. As a consequence, negative weight factors can be removed completely by linearizing the logarithm around one. We consider two forms of such a linearization: in the first, we use the linearization only for values of 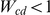, in the second, we completely replace the logarithm by the linearized form (see inset of Fig. 1A):
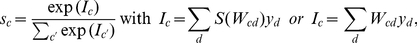
(12)where 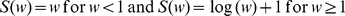. For the linearization we exploited that for normalized inputs the softmax becomes invariant with respect to weight offsets (see Methods). The linear case recovers the conventional linear summation of synaptic inputs, while the logarithmic case is a closer approximation of the optimal dynamics (see Discussion).

The complete description of the final neural circuit is summarized in Table 1. It consists of essentially three elements: input normalization, Hebbian plasticity with synaptic scaling, and softmax competition (see also Fig. 1). Our analysis shows that these elementary models of neural interactions can be approximately optimal for learning on normalized inputs from mixture distributions. Notably, the neural circuit can process any type of un-normalized data as feedforward inhibition projects any stimulus to a subspace on which learning is optimal.

**Table 1:** Learning in neural circuits.

lateral inhibition	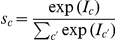
input integration	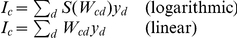
synaptic plasticity	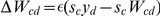
feedforward inhibition	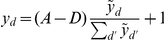

Summary of neural interactions for approximately optimal learning in our model. The function 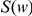 is given by 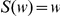 for 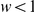 and 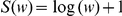 for 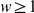 (see Fig. 1A).

It is important to remark that no explicit knowledge about 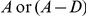 is required at the level of processing neurons, which would be difficult to justify neurally. Instead, synaptic scaling automatically adjusts the weights 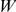 such that the constraint in Eq. 2 is satisfied. This, furthermore, means that synaptic plasticity can follow slow changes of the normalization constant 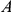, which could be used to further facilitate learning. Formally, manipulating 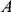 during learning provides a simple implementation for simulated annealing, which is often used to prevent optimization from converging to locally optimal solutions [38], [39]. Alternatively, annealing can be achieved by changing the amount of spontaneous activity in the input layer (see Discussion for neural mechanisms implementing such changes).

Considering the details of the neural circuit and the generative model used here, some aspects of the analytical results presented may not seem very surprising. The similarity between the fixed points for the synaptic weights and the maximum likelihood solution is partly due to the fact that both models fulfill the same constraint, 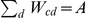, at least approximately. However, this constraint has different origins in the two models: in the neural circuit it is a reflection of synaptic scaling, whereas in the generative model it appears due to the fact that the modeled data is normalized. Along the same lines, the fact that the softmax function emerges as the optimal transfer function for the circuit is somewhat expected, given that the softmax is closely associated with mixture models. However, the arguments of the softmax, 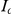, have a particularly compact form in our case, and they can be easily approximated through the integration of afferent inputs to the processing neurons. The compactness of the neural interactions is a direct consequence of the combination of Poisson mixture distributions, normalized inputs and synaptic scaling. Without any of these components, the interactions would be more complicated, or not optimal.

### Optimal learning – numerical simulations

Although we have shown that learning in the neural circuit approximates optimal learning for our data model, several details remain to be investigated. First, it is unclear how close is learning in the neural circuit to the optimum in practice. Second, since real data rarely follows the assumptions of the model exactly, we would like to know how robust learning is in such cases. These questions can only be answered through numerical simulations using either simple artificial data for which the optimal solutions are known, or realistic inputs from a standard database.

#### Artificial data

**Figure 2:**
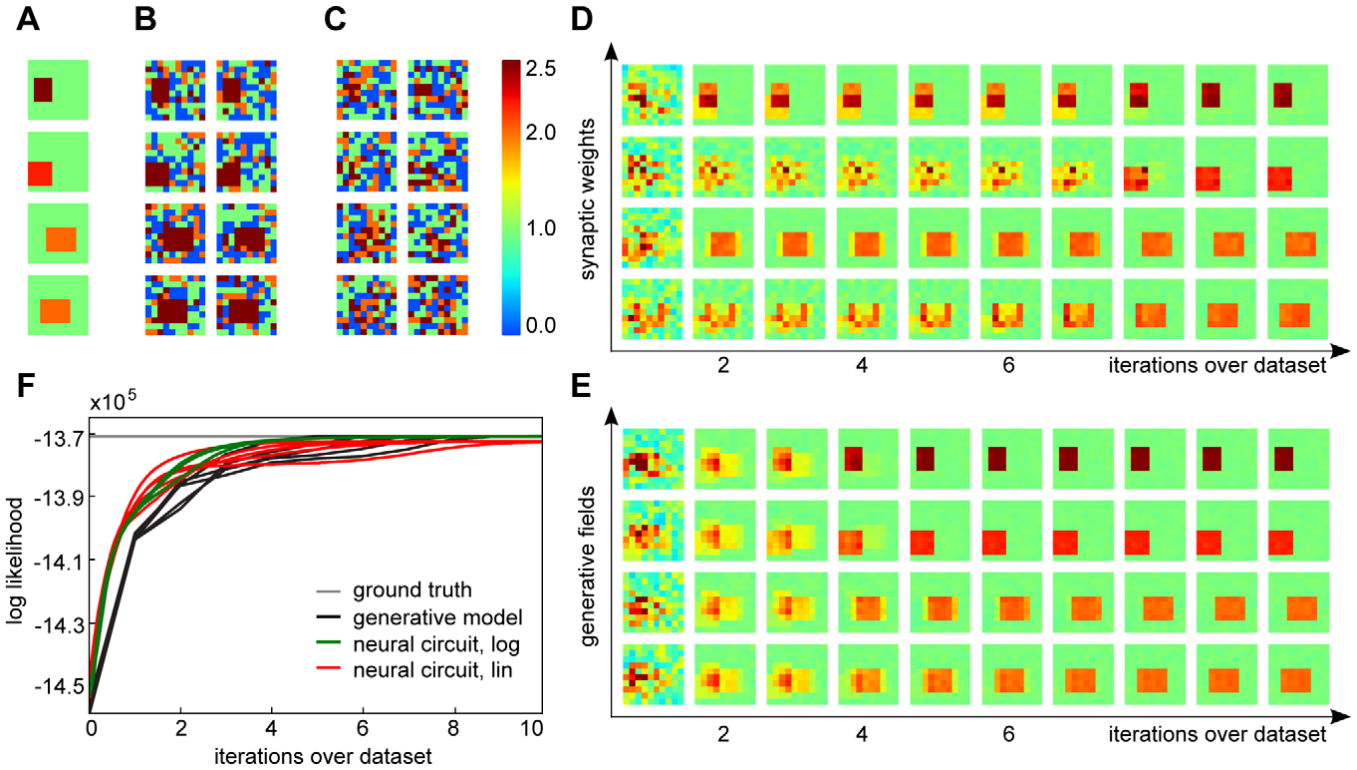
Learning on artificial data. (A) An example set of generative fields 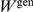, for 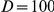 (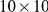 pixels). Due to the normalization, different rectangles have different pixel intensities (displayed here for 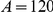). (B) Some examples of generated data for the same rectangles as in (A) with normalization constants 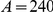. (C) Same examples with 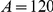. Very high intensity values were truncated to improve visibility. (D) The evolution of synaptic weights during learning in the neural circuit (linear case) if data as in (C) was used. (E) Evolution of the generative fields using EM algorithm for the same data. (F) Likelihood changes during learning for the neural circuit (both versions) and EM; learning used 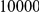 inputs from the classes shown in (A) with 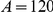. Different lines of the same color mark individual runs with different random initial conditions.

We consider an artificially generated data set, for which ground truth about the input distribution is available. In particular, input stimuli are generated by the normalized mixture model (Eqs. 6 and 7), using generative fields 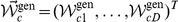 in the shape of partially overlapping filled rectangles, with background values set to one, see Fig. 2A. The degree of overlap of the rectangles and their relative sizes determine the difficulty of the task. Note that all data will be visualized two-dimensionally, i.e., we show inputs 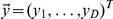 and the synaptic weights of a neuron 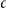, 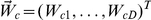, as 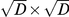 pixel images.

Some example data, generated with 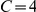 classes, 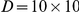, and different normalization constants are shown in Fig. 2B,C. High values of 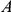 (Fig. 2B) correspond to a high signal-to-noise ratios, while low values of 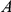 (Fig. 2C) result in very noisy data. In annealing terms, a low 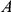 corresponds to a high temperature, which makes the system more flexible to explore the space of possible parameters and helps avoid local optima. Here, we keep 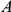 fixed during learning and optimize its value for best performance (for this data 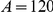, with performance deteriorating for values larger than 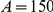).

We generated 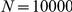 data points with generative fields as those in Fig. 2A, which we use to learn the weights in the neural circuit and for the EM parameter optimization (the detailed setup for these experiments is described in the Methods). The evolution of the synaptic weights during learning for an example run in the linear neural circuit is shown in Fig. 2D. The corresponding evolution of the generative fields using EM optimization is shown in Fig. 2E. Both converge after about 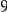 iterations over the whole data set (we repeat the input data in the neural circuit as well, for a closer match to EM). Also the neural circuit with log-saturation of inputs shows a behavior very similar to EM (not shown). For a more quantitative comparison of learning in the two systems, we use two measures: the likelihood of the input data under the model, given the learned model parameters, and the percentage of trials which converge to the global optimum.

First, the evolution of the likelihood during learning is shown in Fig. 2F for the different versions of the model. During learning, the circuit parameters improve continuously to a value close to the likelihood of the ground-truth parameters and therefore close to the optimal value for the data. For comparison, the same plot also shows the likelihood values during EM optimization, which converges to the optimum with a small amount of overfitting (hardly visible in the figure), same as the neural model with log-saturating inputs. The great similarity between the obtained likelihoods confirms the high accuracy of the approximations used in the neural circuit with log-saturation. Likewise, the neural circuit with linear input summation converges to close to optimal likelihood values. The slightly lower final values are attributed to the stronger effect of the fully linear approximation. Second, regarding the recovery of global vs. locally optimal solutions, learning in the circuit converges to the approximately optimal solution for normalized data in most of the runs. Specifically, neural learning in the simple neural circuit recovers the global optimum in 86 of 100 runs, while the log-saturating version further improves this number to 97 of 100 runs; for comparison, EM learning converges to global optima in 96 of 100 runs.

#### Realistic data

**Figure 3:**
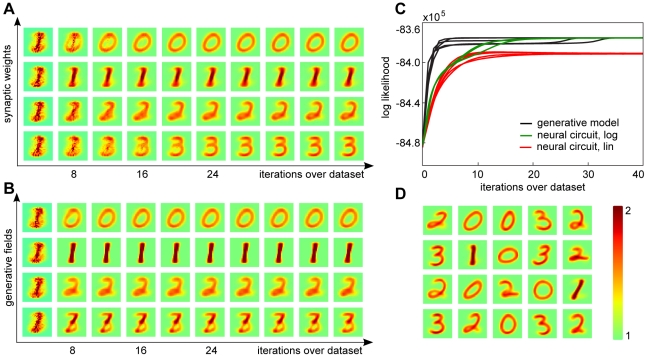
Learning on more realistic data. (A) Evolution of synaptic weights in the neural circuit on inputs from the MNIST database. (B) Evolution of generative fields using EM on the same data; for both input data consisted of 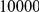 data points from the digit classes 0 to 3 with normalization 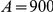. (C) Changes of the likelihood during learning for the neural circuit (both versions) and EM. (D) Synaptic weights learned by the circuit (linear version) on the same data but with five times more processing neurons.

We have seen that learning in the neural circuit shows close to optimal performance when the input data is generated according to the assumed mixture model. Real data, however, does not match the assumptions of our model exactly. If we take, for instance, the MNIST dataset of handwritten digits [40], [41], a standard dataset for classification, differences between different items from the same class arise from different writing styles for the same digit. Although writing style variations are not modeled explicitly, we expect the stochasticity modeled by Poisson noise to capture these variations at least partially, allowing for robust learning in this setup. Hence, we use this dataset for learning in our model. We start by normalizing the data by feedforward inhibition (Eq. 11), after which learning proceeds as for the artificial data (see Methods for details). The emerging weights in the neural circuit (linear case) and the corresponding generative fields for an example run using digits ‘0’ to ‘3’ are shown in Fig. 3A,B. As can be observed, both the neural circuit weights and the learned generative fields of EM converge to represent individual digits.

A quantitative analysis of the learning outcomes is more difficult in the case of realistic inputs, as we no longer have access to ground-truth information. Nevertheless, we can still compare the likelihood values during learning. Fig. 3C shows the evolution of likelihoods for both circuit models and for EM. As can be observed, the likelihood values for both the neural circuit and EM again continuously increase. As before, the log-saturating circuit and EM converge to virtually identical likelihood values. For the linear circuit, there is again a gap, slightly more pronounced this time (but also note the finer y-axis scale). Still, the neural circuit is very similar to EM in representing individual digits (Fig. 3A,B).

In general, unsupervised learning in the circuit and EM try to cluster the available data as well as possible, regardless of the ‘true’ class labels. In particular, because of similarities between different digits, the emerging generative fields do not necessarily reflect the digits' class distinction. If we use the full MNIST dataset and ten processing neurons, similar images from different classes, e.g. a ‘3’ and ‘8’ with similar slant, are often clustered together. As a consequence, the neural circuit and EM usually fail to represent all classes. A straight-forward solution for this problem is to increase the number of neurons in the processing layer, which allows for a finer grain representation of the inputs. In such an overcomplete setting, learning can successfully represents all classes. Furthermore, when several neurons learn the same digit, they represent different subclasses (e.g., different slants for ‘3’), as shown in Fig. 3D. In the following, we show that these emerging representations can be used by a higher neural processing layer for efficient classification.

### Higher level processing – a classification task

Until now, we have evaluated the effectiveness of learning by measuring how well the final weights can describe the data (formally, the data likelihood under the generative model). Alternatively, we could ask how useful the emerging input representation is for performing higher level tasks in downstream circuits. The performance for such tasks can give a measure of learning quality that is more independent of specific assumptions about the input statistics. Moreover, such alternative performance measures become a necessity when comparing learning on normalized versus un-normalized data, as done in the following section. Since likelihoods are well-suited measures of learning performance only when computed using the same data, no such comparison is possible when trying to asses the benefits of normalization.

For the MNIST dataset, a natural task is classification, which has been extensively investigated in the literature, both in neural models and using purely functional approaches (e.g., [42]–[44]). Note, however, that the type of classification relevant for biological systems differs from the generic classification in several aspects. Perhaps most importantly, stimuli processed by neural circuits usually come without explicit labels. For instance, most visual stimuli we process are not accompanied by labels of the visual objects that caused them. However, during development we are provided (directly or indirectly) with the meaning of objects for some stimuli. In order to classify inputs accordingly, the model needs to have access to at least some stimuli 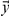 for which the class membership (label) is known. These labels can then be used to associate the representations in the lower processing layer (obtained by unsupervised learning) with the corresponding class; for instance, all writing styles of a hand-written ‘2’ with digit class ‘2’. Having an overcomplete representation of the data becomes critical for the system to work in this setup. As we have seen in previous numerical experiments, learning with MNIST data yields representations of different classes of hand-written digits. Because of different writing styles, the variations of all patterns showing the same digit are too strong to allow for a representation of all digits with one class per digit. However, as already shown in Fig. 3D, with more neurons than classes, the emergent representation successfully captures all digit classes, with different neurons representing different writing styles (the more units, the more detailed the representation of different writing styles).

**Figure 4:**
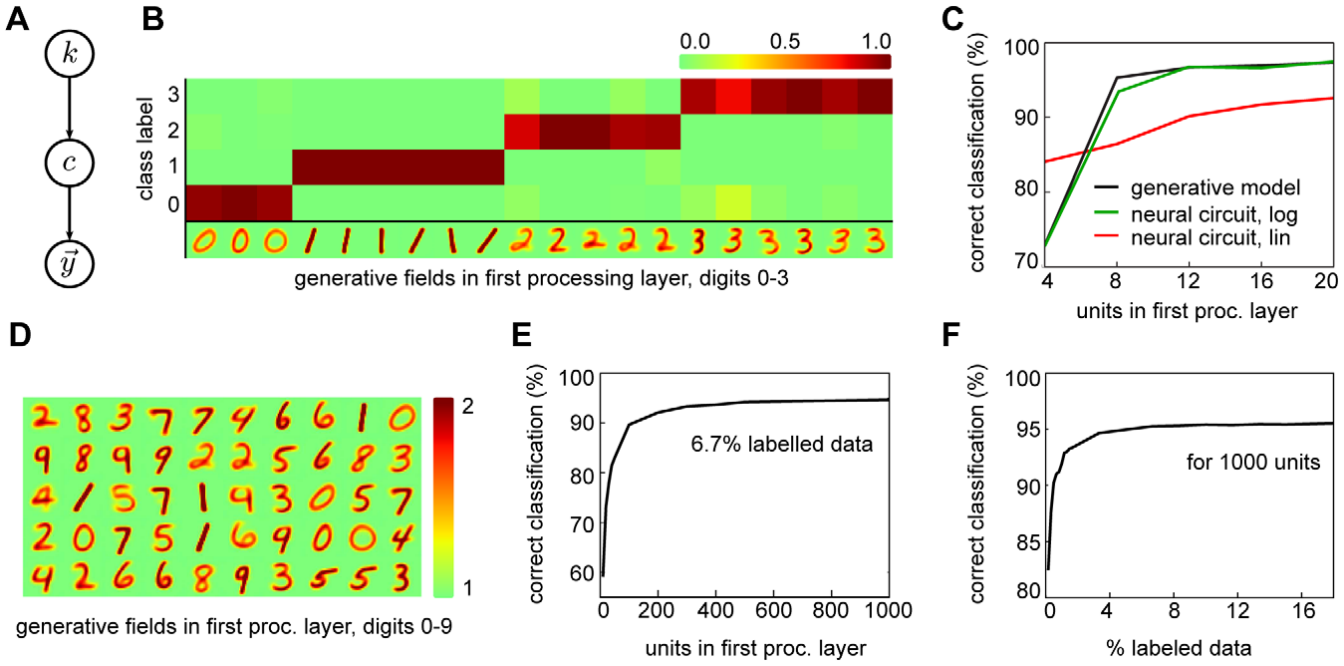
Classification of MNIST inputs. (A) A graphical model linking the representations in the first processing layer, learned in an unsupervised setting, to class labels 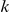 in a second processing layer. (B) The assignment of the learned generative fields to digit classes obtained using 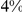 of the labels in the set of 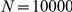 training inputs (subset of MNIST with classes 0 to 3). (C) Classification rates after training for the neural circuit (both versions) and EM on the MNIST test set (classes 0 to 3). (D) Generative fields for 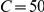 classes for EM trained on the full MNIST training set (10 digit types). (E) The classification rate based on the generative fields learned by EM for the full MNIST data set (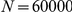). Rates are plotted as function of the number of units in the first processing layer. For the results 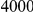 labels of the training set were used (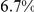). Error bars (10 runs) were, in general, too small to be visible: for 100 units, different runs divert from the mean classification rate of 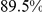 by less than 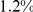; for 300 units by results diverted by 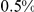; and for 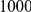 units diversions were at 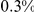. (F) Classification performance as function of the amount of labeled data used for learning in the second processing layer, for 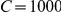 units. As in (E) error bars were, in general, too small to be visible.

For classification, we extend the neural circuit to include an additional processing stage that makes use of the previously learned representation for assigning class labels. As done for the first processing layer, we formulate the classification process probabilistically, using a generative model assuming that a digit type 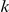 generates different writing styles (Fig. 4A). This allows us to derive a probabilistic procedure for classifying a given input stimulus (see Methods). The focus here is assessing the utility of the first layer representation for higher level computations rather than the neural implementation of this later processing stage. Still, we can note that the dynamics of the second layer shares several features with the first layer model: the neural dynamics have a simple dependency on a weighted sum of incoming inputs (see Methods), and the inputs themselves are normalized (because of the softmax), suggesting this type of computation could be implemented in a neurally plausible circuit.

To illustrate classification based on the representations learned unsupervised, we first consider stimuli representing digits of types ‘0’ to ‘3’. For this data, the representations learned by unsupervised learning in the first processing layer (with 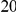 units) is shown in Fig. 4B (bottom row). We label these representations using 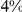 of the data used for training (i.e., we use the labels of 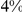 of the training data). The probability distribution for the map between first layer representations and class labels is shown in Fig. 4B (computed using Eq. 34, see Methods), demonstrating a close to perfect assignment of representations to digit classes. For a quantitative analysis of this match, we can measure the classification performance of the system for a test dataset (i.e., for data not used for training; see Methods for details). For the four digit dataset, the classification performance as function of the number of neurons in the first processing layer is shown in Fig. 4C. For both the neural circuit and EM optimization classification performance increases with the number of units. As can be observed, the neural circuit with log-saturating synaptic efficacies shows virtually identical classification rates to EM learning. Likewise, the neural circuit with standard linear input summation shows a good classification performance, even slightly better for the complete case (four digit classes and four processing neurons). In an overcomplete setup, the rate of successful classifications is still high (e.g., around 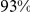 for the five times overcomplete setup), though a bit lower than for the log case and EM.

So far, we have used classification performance as an additional measure for the quality of learning in the circuit. However, the setup is interesting from a functional perspective as well, since it allows for relatively high rates of correct classification using a very limited amount of labeled data. Fig. 4E shows classification performance for different degrees of overcompleteness in the processing layer if normalized EM is applied to the full MNIST data (we use EM here as it can be efficiently scaled-up to the size of the full MNIST dataset; see Methods). As before, classification performance increases with an increasing number of units and with the number of labels used for classification (see Fig. 4E and Fig. 4F, respectively). Importantly, a small percentage of labels is already sufficient to obtain almost the same classification performance as when using all labels. For instance for 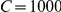 processing units we obtained a performance of 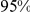 correctly classified stimuli using just 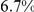 of the MNIST labels. For rates above 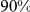 less than 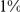 of labels were sufficient. Moreover, performance in our model is comparable to that of state-of-the-art methods, such as deep belief networks (DBN; [42]). Using all the labels, the performance of DBN reaches 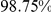 [42], but with a much more complex circuit (two processing layers and an associative memory), several learning mechanisms, and after the tuning of many free parameters. In contrast, learning in our model is very straightforward, with very few free parameters (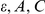), and requires just few labeled inputs. These properties seem particularly desirable in the biologically relevant setting.

### Functional benefits of input normalization

Even if we assume that synaptic scaling is unavoidable to guarantee stability during Hebbian learning, it is still unclear why the system would need feedforward inhibition, or, more in formal terms, what are the benefits of learning using normalized data. This question can be addressed at two levels. First, at an abstract level, we can ask how different are the outcomes of optimal probabilistic learning when using unconstrained versus normalized data. Second, in neural terms, we can ask how learning changes when blocking feedforward inhibition in the neural circuit.

**Figure 5:**
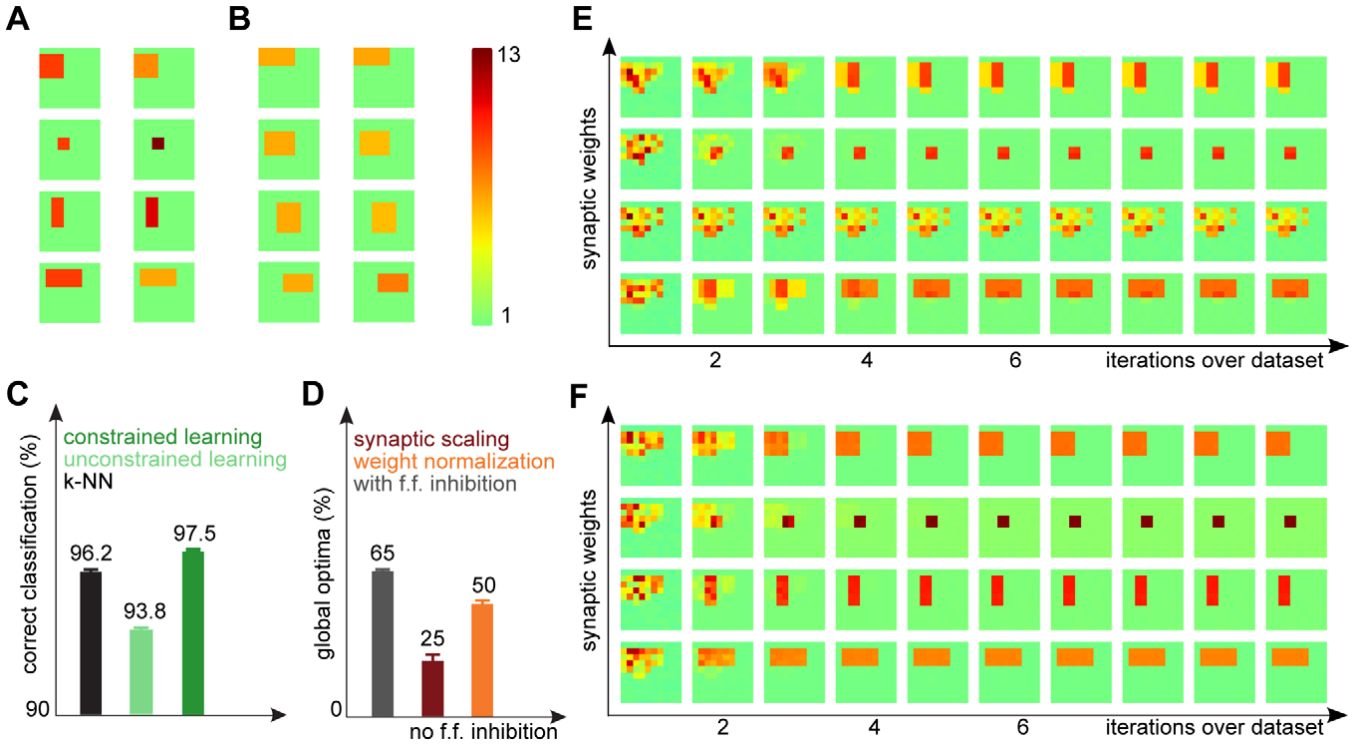
The contribution of feedforward inhibition and synaptic scaling to learning. (A) An example set of generative fields for unconstrained (left column) and normalized (right column) data. The overall average intensity across all fields is constrained to facilitate the comparison of learning with different models. (B) Same as before, but with rectangles of similar sizes. (C) Rate of correct classification for optimal learning with constrained vs. unconstrained data. (D) Rate of convergence to global optima when learning from (un)constrained data with the linear network model, when weights are constrained either by local synaptic scaling, or through explicit normalization. All estimates are computed out of 100 trials. (E) Evolution of the synaptic weights when synaptic scaling is implemented either by synaptic scaling or (F) as instantaneous weights normalization, for an example run.

To answer the first question, we use our generative model approach to compare the optimal learning dynamics for data that is, or not, normalized (this difference will depend on the relative size of different stimuli; compare Fig. 5A and B). Formally, we construct an analog mixture model for un-normalized data, and derive optimal learning for this model. The analysis yields a similar set of update rules (see Methods, Eqs. 26 and 27), which we can use for unsupervised learning with similar (but un-normalized) data. Because the two learning procedures use different data, comparing them is nontrivial. While for data generated according to the assumed probabilistic model we can still use the percentage of trials converging to the optimum as a performance measure, comparison becomes very difficult for the digits data. Since the likelihoods are no longer comparable (because they are estimated from different data), we can only rely on the classification rates for estimating the quality of the learned representations in this case.

We compare the performance of the two learning procedures for the same two datasets described above. For the blocks dataset, learning performance is not significantly different in the two cases (not shown), probably because the task is too easy to be able to differentiate between the two learning procedures. The results for the digits are shown in Fig. 5C. The unconstrained learning procedure yields worse performance than the constrained case; the difference may seem small in absolute terms, but the classification rate for the unconstrained case is worse than the outcome of k-nearest-neighbour (k-NN) classification, which we may view as a lower bound for task difficulty. In itself, this result is not sufficient to prove that learning from normalized data is generally useful for unsupervised learning. Since we can only estimate learning performance indirectly, through the classification rates, it may be that data normalization improves classification in general, by removing task irrelevant variability, without having any specific benefit for learning per se. If this were the case, then we should observe a similar performance improvement for the normalized relative to the unnormalized data when using a standard classifier, such as k-NN. This is however not the case; on the contrary, for k-NN performance decreases to 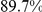 (from 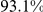) after data normalization, suggesting that the benefits of normalization are restricted to learning procedures that explicitly exploit this property, as does learning in our model.

For the neural circuit, the utility of the interaction between feedforward inhibition and synaptic scaling is further emphasized. When blocking feedforward inhibition (practically, this means using unnormalized stimuli as inputs to the circuit) the linear circuit converges to represent all classes very rarely, much less often than when feedforward inhibition is active in the circuit (Fig. 5D, compare grey and red bars). In principle, since the neural circuit approximatively implements optimal learning for normalized data, one could expect that performance should be similar to that obtained by constrained EM with un-normalized data, which is indistinguishable from that obtained when learning from normalized data. So why is there a the big difference in performance in the case of the neural circuit? The critical difference between EM and the network is that synaptic scaling only enforces the constraint of the weights through its (normalized) inputs. If the incoming stimuli are not normalized, the sum of the weights is not guaranteed to converge at all (Eq. 4 does not apply). This intuition is confirmed by the fact that when replacing synaptic scaling by an explicit weights normalization (see Methods) learning evolves similarly to the case when feedforward inhibition is active. These results suggest that feedforward inhibition is critical for correctly learning the structure of the data when the weights are constrained by biologically plausible synaptic scaling.

## Discussion

Our results reveal a close connection between feedforward inhibition and synaptic scaling, which could be important for cortical processing. We have shown that an elementary neural circuit with lateral inhibition, Hebbian plasticity and synaptic scaling can approximate optimal learning for normalized inputs. Furthermore, although our analysis demonstrates the approximate equivalence between learning in the neural circuit and the optimal theoretical solution only when inputs are generated by normalized mixture distributions with Poisson noise, numerical simulations using realistic data show that close to optimal learning is possible even when the inputs do not match these model assumptions exactly. Importantly, optimal learning is an outcome of a synergistic interaction between input and weight normalization, and learning is much less effective in absence of any of the two.

The mechanisms required for optimal learning in our model circuit have close correspondents in biology. First, the type of input normalization used in our model has been observed in both hippocampus and the cortex [11]. It involves a population of fast-spiking inhibitory neurons that deliver relatively homogeneous inhibition to the pyramidal cells. For a more detailed map of our model onto this circuit, we assume, in first instance, that the normalized version of the stimulus is explicitly represented in one layer, which then projects onto the processing layer. Alternatively, it is imaginable that the normalized stimuli could only be available in implicit form, without the need for an additional input layer; this would, however, require some corrections to the Hebbian learning rule, since the presynaptic term would depend on the input scale in this case. Second, learning in the circuit takes a simple local form, which has natural biological correspondents. In particular, for the linear approximation for synaptic currents, learning involves simple Hebbian plasticity and multiplicative synaptic scaling. The map to biology is somewhat more difficult for the model with logarithmic saturation of synaptic efficacies. This would translate in an unconventional type of weight-dependent Hebbian learning, and more complex additive synaptic scaling. Although there is some data on weight-dependent correlation learning [45] and additive synaptic scaling has been reported in some systems [46], the experimental evidence clearly favors the linear approximation for synaptic currents. The logarithmic version is nonetheless important, as the closest approximation to the optimal solution with bounded excitatory input currents. Moreover, it enables us to quantify the effect of the approximations in the linear model and hence to explain the difference in performance of the neural circuit relative to the theoretical optimal solution. Lastly, optimal learning requires a lateral interactions between the processing neurons, mathematically described by the softmax function. Due to its importance for competitive learning, different circuit models giving rise to softmax or softmax-like competition have been investigated previously [26], [32]–[34], [36], [37], typically involving lateral inhibitory networks with uniform connectivity onto the excitatory population. Experimentally, evidence for such lateral inhibition has recently been reported, for instance, in primary sensory cortex, where feedback inhibition relies on broadly tuned interneurons, that integrate information from pyramidal cells with diverse stimulus preference [47], confirming earlier anatomical observations (see [48] for an overview).

We have seen that the normalization constant plays an important role during learning, as it controls the sharpness of the posterior distribution which in turn influences the frequency to converge to locally vs. globally optimal solutions. Learning outcomes can be improved by annealing this parameter throughout learning. Biologically, several neuromodulators are known to affect the response properties of inhibitory neurons [49] in a way that would effectively change the normalization constant. Alternatively, the modulation of background noise can affect neuronal gain in cortical neurons [13], [15], which, in the model, has similar effects (since both change input contrast). It is tempting to speculate that the effectiveness of learning can be manipulated by systematic changes in background current or in the concentration of neuromodulators, such as acetylcholine, dopamine or noradrenaline [49], [50]. This would suggest that experimentally manipulating the concentration of these substances in the cortex should have predictable effects on learning efficiency, although these may be difficult to dissociate from other effects of such manipulations on arousal or attention [51].

Activity normalization is ubiquitous in the cortex. In particular, divisive normalization – when a neuron's response is rescaled as function of that of its neighbors – has been reported for a variety of sensory systems, from visual [52]–[54], to auditory [55], [56] or olfactory [57]. Correspondingly, a range of functions have been attributed to such normalization. It could optimize the representation of visual inputs in primary sensory areas [58], [59], facilitate the decoding of information from probabilistic population codes [60], explain attentional modulation of neural responses [61], or implement multi sensory cue integration [62]. While the form of normalization considered here is not equivalent to standard models of divisive normalization (which typically assume an L2 norm) and seems to have different neural substrates [63], several interesting parallels can be drawn with these models. In particular, we can view feedforward inhibition as a way to constrain the space of representations, similar to [59]. However, instead of asking how normalization affects the information that can be encoded in the population as a whole, we investigate how activity normalization constrains learning in neurons receiving it as inputs.

The simple, biologically plausible neural circuit proposed here achieves robust, close to optimal unsupervised learning through the interaction between feedforward inhibition and synaptic scaling. Moreover, the two are mirror processes, which need to work together for Hebbian learning to yield efficient representations of the inputs to the network. Since the type of neural mechanisms involved in our model can be found throughout the cortex, it is tempting to suggest that the interaction between feedforward inhibition and synaptic scaling could be a general strategy for efficient learning in the brain.

## Methods

### Evolution of weights – details

Learning in the neural circuit consists of iterative applications of Eq. 1 and Eq. 3 to normalized input data 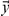, which is drawn identically and independently from a stationary distribution 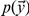. To facilitate numerical analysis, we assume that learning uses a finite dataset of 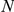 stimuli, presented repeatedly to the network in random order. In the limit of large 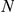, this procedure becomes equal to drawing a new sample from 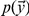 each time.

For the learning dynamics Eqs. 1 to 3 we can show that the synaptic weights 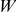 approximately satisfy Eq. 5 at convergence. The approximation holds for small learning rates 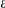 and large numbers of inputs 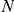. Large learning rates would bias learning towards recent inputs. A small dataset would introduce a large sample bias such that averages across the dataset would be significantly different from expectation values w.r.t. the distribution 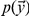 in Eq. 5. For the derivation nested terms scaling with 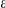 and applied 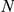 times have to be considered, which requires a series of rather technical approximations. We, therefore, present the essential steps here and provide the details as supplemental information (Text S1).

For the derivation, we consider learning after convergence, i.e., after the changes of 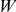 have reduced to changes introduced by random fluctuations due to online updates. For small 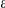 these fluctuations are small. Let us denote by 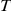 an iteration step after which only such small fluctuations take place. After iteration 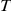 we can assume the weights 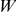 to have evolved to satisfy 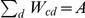 for all 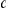 (which follows from Eq. 4). For small 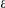 the learning dynamics (1) to (3) is approximated by changing the weights according to 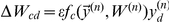 followed by an explicit normalization to 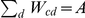. More compactly, we can write:
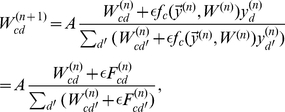
(13)where 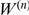 denotes the weights at the 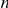
th iteration of learning, and 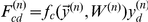.

We now consider another 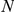 learning steps after iteration 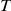, i.e., we iterate through the inputs once again after learning has converged. By applying the learning rule (13) iteratively 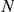 times, the weights 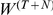 are given by (see Text S1):
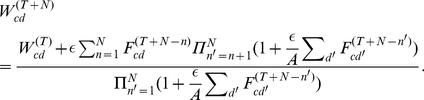
(14)The right-hand-side can now be simplified using a sequence of approximations, all of which are based on assuming a small but finite learning rate 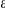 and a large number of inputs 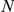. Below we present the main intermediate steps of the derivation and list the approximation used for each step:
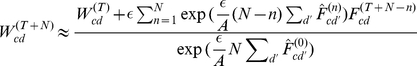
(15)

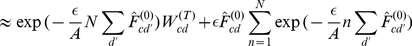
(16)

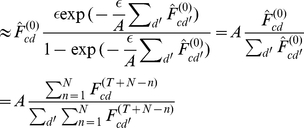
(17)where 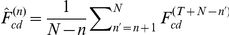 (note that 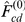 is the mean of 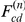 over 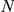 iterations starting at iteration 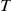).

For the first step (15) we rewrote the products in Eq. 14 and used a Taylor expansion (see Text S1):
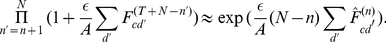
(18)


For the second step (16) we approximated the sum over 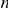 in (15) by observing that the terms with large 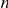 are negligible, and by approximating sums of 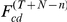 over 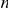 by the mean 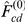 (see Text S1). For the last steps, Eq. 17, we used the geometric series and approximated for large 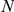 (see Text S1). Furthermore, we used the fact that for small 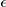, 
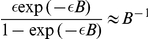
 (which can be seen, e.g., by applying l'Hôpital's rule). Finally, we back-inserted the definition of 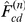 for 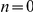.

By inserting the definition of 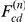 into (17) and by applying the assumption that the 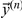 are drawn from a stationary distribution 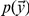, it follows that:
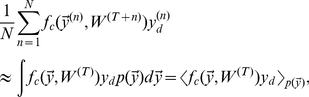
(19)yielding the final expression:
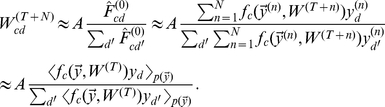
(20)For Eq. 19 we used the initial assumption that the weights have converged, i.e., that 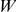 remains approximately unchanged after 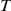. If the same assumption is applied to Eq. 20, we obtain Eq. 5.

Note that although we have applied a number of different approximations during this derivation (compare [64] for proof sketches of some of them), each approximation is individually very accurate for small 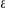 and large 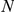. Eq. 5 can thus be expected to be satisfied with high accuracy in this case; subsequent numerical simulations for a specific choice of the transfer function 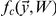 confirm such high accuracies.

### Derivation of the EM update rules

Given a set of 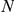 inputs drawn from an input distribution 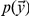, optimal generative model parameters 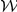 can be found by optimizing the likelihood: 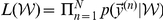. A frequently used approach to find optimal parameters is expectation maximization (EM) [24], [25]. Instead of maximizing the likelihood directly, EM maximizes a lower-bound of the log-likelihood, the free-energy:
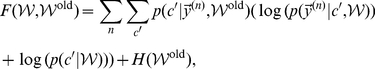
(21)where 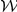 and 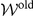 are the newly computed and previous parameters of the generative model, respectively, and where 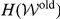 is an entropy term only depending on the previous parameters. To optimize the free-energy, EM alternates between two steps – the E-step and the M-step. First, in the E-step, the parameters are assumed fixed at 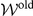 and the posterior 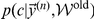 is computed for all data points 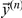. Second, in the M-step, the model parameters are updated using these posterior values. Note that for more general models, computations of expectation values w.r.t. the posteriors are considered part of the E-step. For mixture models such expectations are tractable operations, and we, therefore, often use E-step and computation of the posterior synonymously.

M-step solutions can be found by setting the derivative of the free-energy w.r.t. 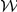 to zero. Applied to the concrete model of normalized input given by the mixture model (Eq. 6), we have to optimize the free-energy under the constrained of normalized weights: 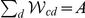. We can satisfy the constraint by using Lagrange multipliers for the derivatives and obtain:
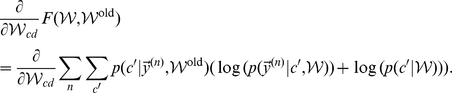


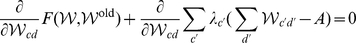
Expanding the expression for the free energy and computing the partial derivatives gives (all 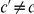 drop out):
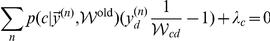


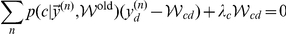
Taking the sum over 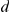 and applying the constraint 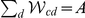, we can rewrite the above expression as:
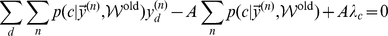


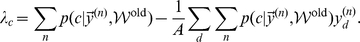



Inserting the value of 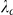 computed above and solving for 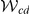 yields:
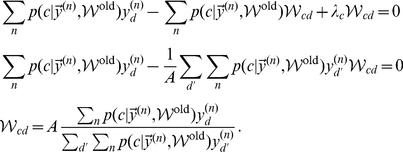
(22)For the normalized mixture model (Eq. 6 and Eq. 7), the posterior probability 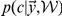 can be computed directly. By inserting the Poisson noise model and constant priors, 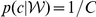, and by using the constraint on the weights, the posterior can be simplified as follows:
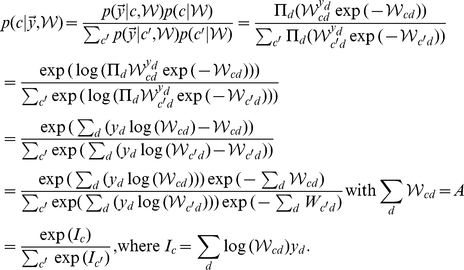
(23)Note that the specific combination of normalization constraint and Poisson noise results in the final compact form of the posterior. The E-step consists of computing these posteriors for all inputs 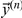


To summarize, putting together 22 and 23, E- and M-step for our model of normalized data are given by:
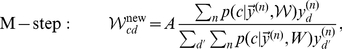
(24)

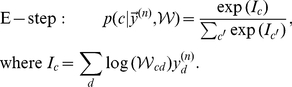
(25)


#### Update rules for unconstrained learning

In order to investigate the effects of feedfoward inhibition on learning, we need to derive the optimal learning rules for a mixture model that does not assume normalized generative fields. The derivation is very similar to the one above and more conventional because no Lagrange multipliers are required for enforcing the normalization constraint. The E- and M-step equations for the unconstrained case are given by:
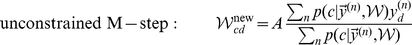
(26)

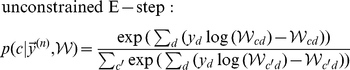
(27)Note that enforcing the weight normalization 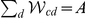 in the above expression recovers the expression for the constrained EM before.

### Linearization of input integration - details

To further simplify the computation of the posterior in Eq. 8, first note that due to normalized input, 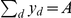, the posterior computations remain unchanged for any offset value 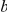 for the weights:
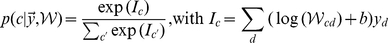
(28)

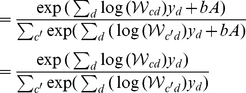
(29)

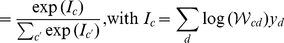
(30)If we use an offset of 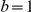 we can approximate 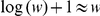 by applying a Taylor expansion around 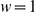. If we use the linear approximation for values 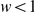 only, we obtain the function 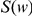 in Eq. 12. For data with 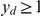 as enforced by Eq. 11, the weights will converge to values greater or approximately equal to one, which makes 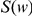 to a very accurate approximation. If we use the linear approximation for all values of 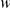, we obtain the conventional linear summation in Eq. 12.

### Higher level processing – details for classification

In order to use the representation of pattern classes in the first processing layer for classification, we consider the hierarchical generative model in Fig. 4A. The model assumes the patterns to be generated by the following process: First, choose a pattern type 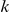 (e.g., 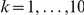 for ten digit types), second, given 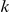 choose a pattern class 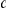 (e.g., different writing styles), and, third, given 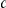 generate the actual pattern 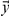 (with added noise). For the generation of pattern types 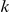 we assume flat priors 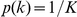, i.e., we assume that each type is equally likely.

Under the assumption that the data is generated by the model, optimal inference is given by computing the posterior 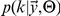, where 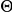 are the parameters of the model. By using the form of the graphical model in Fig. 4A, we obtain:
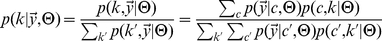
(31)


The probabilities 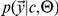 are given in Eq. 6 (right-hand-side). To estimate the probabilities 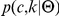 let us first define the sets 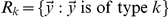 and let us assume these sets to be disjoint (no overlap). In this case we obtain:
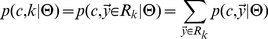
(32)

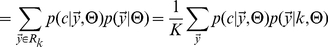
(33)

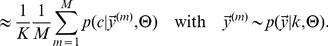
(34)Together with Eq. 31, the estimate for 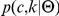 allows for a convenient way to approximate the posterior 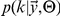 using input labels:
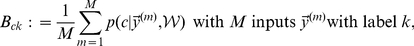
(35)

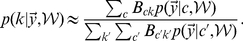
(36)


That is, we can compute the values 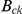 using 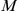 labeled inputs 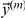 for each type 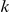. Having computed all 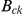, the approximate posterior given an unlabeled input is given by Eq. 36. Few labeled inputs can be sufficient to get good estimates for 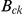 and thus for the posterior computation (compare Fig. 4B). Note that Eqs. 35 and 36 can only be regarded as approximations for optimal classification because of the assumptions made. However, they serve in providing good classification results (see Results), the 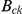 can conveniently be computed after unsupervised learning, and, the 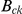 can be interpreted as weights in a neural processing context.

After unsupervised learning and computation of 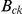 using Eq. 35, an input 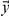 is assigned to the digit type 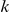 with highest posterior using Eq. 36. If the assigned type matches the true label of 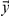, the input is correctly classified. Note, in this context, that our approach would also allow for a quantification of the classifications' reliabilities by comparing the different values of 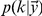.

Finally, note that the setting of few labeled inputs among many unlabeled ones is typical for semi-supervised learning. Algorithms for semi-supervised learning usually take labeled and unlabeled data into account simultaneously. As we focus on unsupervised learning and use the labels for a second stage of classification, we avoided to refer to our approach as semi-supervised.

### Simulation details

For all simulations we initialize the weights 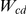 with the mean pixel intensity 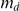 averaged over all data points, with some additive uniform noise:
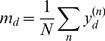
(37)

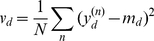
(38)

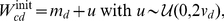
(39)where 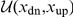 is the uniform distribution in the range 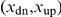.

#### Artificial data

We generate a dataset of 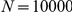 images using our mixture model. The generating parameters 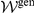 (Eqs. 6 and 7) used are of the type as shown in Fig. 2A, normalized with 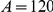. More specifically, the data generating process involves first choosing a class 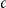 from the prior, and then applying Poisson noise to the corresponding generative field 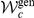. We randomly create a new set of parameters 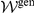 for each trial, each consisting of 4 fields with block sizes varying in the interval 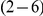 pixels, constrained such that the degree of overlap between any two blocks is in between 1 to 50%. The resulting dataset is repeatedly presented to the neural circuit, with the order of the data points permuted for each block. Learning in the neural circuit proceeds according to Eq. 3, with the learning rate 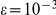. For the corresponding EM learning the same parameters and the same data is used.

#### Realistic data

For the numerical experiments shown in Fig. 3, we used 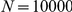 data points of the digits ‘0’ to ‘3’. These data points are subsamples of the MNIST data set to guarantee equal representation of each digit (note that for the numerical experiments in the section ‘Higher level processing’ we do not use subsampling). We normalized the resulting dataset using Eq. 2. Note that this ensures that each input is normalized exactly while each of the artificial inputs used before was normalized approximately. Another distinction is that the new input images no longer have background noise. For the MNIST data we used with 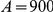 a larger normalization constant than for the artificial data, which is needed due to the higher input dimensionality (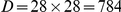). Learning proceeds in the same way as for the artificial data before; the learning rate of both neural circuit models (the log case and the linear case) is 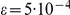, chosen such that the number of iterations needed to converge is roughly the same as the number of EM iterations. For the overcomplete setting shown in Fig. 3D, we ran the experiment with 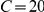 neurons in the processing layer; all other parameters were the same as before.

#### Learning and higher processing on the full MNIST dataset

Since we want to estimate the best possible result for MNIST digits classification, we apply annealing while learning the generative fields. For computational reasons, we can only use the EM algorithm for these results because EM can be executed on arrays of linear processors much more efficiently: the batch of 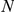 data points can be subdivided into smaller batches and distributed to the array of processors. The number of processors can be chosen such that each small batch can be stored in memory (we used up to 360 processing cores for the MNIST data). The E-step can then be executed in parallel, the results are collected, and the parameters are subsequently updated once per iteration across the batch. While neurally plausible, the online learning of the neural circuit requires an update of the parameters once per input. The parallelization approach for EM is therefore not applicable and learning with hundreds of processing neurons becomes impractically slow. Note, however, that with inherently parallel hardware such as VLSI or FPGA, neural learning could be made very efficient, but the application of such technologies would go beyond the scope of this paper. The neural circuit learning is thus only used with a limited number of neurons (Fig. 4B, C).

For the results shown in Fig. 4D, E, F we started the EM algorithm with 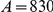 and linearly increase it over 80 EM steps to 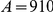. When estimating the classification performance on the MNIST test set, training uses the full MNIST training set, in which the samples are not exactly distributed equally among the digits. In contrast to the numerical experiments with data points from digits ‘0’ to ‘3’ (Fig. 3), we do not subsample the MNIST learning set. Applying subsampling would mean to use indirectly the knowledge of the labels of the data points. Since we apply pure unsupervised learning, we did not want to use this knowledge. The actual performance estimate uses the MNIST test set [40]. Given an input 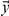 of the test set, we determine the digit type according to Eqs. 35 and 36.

#### Comparison with other methods on MNIST classification

More than a decade of research on MNIST data classification has generated a large body of literature. However, basically all reported approaches are fully supervised (see [40]), i.e., they are using all labels. Many approaches, furthermore, use a larger training set by extending the MNIST training set with adding transformed versions of its inputs. On the original MNIST data, and thus on the same data as used for our systems, deep belief networks (DBN; [42]) achieve 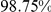 by using all labels. For extended training sets or with systems using build-in transformation invariance [43], [44] still higher classification rates can be achieved (above 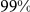). For a baseline comparison with our results, we ran a k-Nearest-Neighbor (k-NN) algorithm; we used the L3 norm for k-NN, since this is known to yields slightly better performance on MNIST compared to the more traditional L2 norm [40]. While such a classifier is very close to the state-of-the-art on extended MNIST training sets (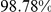, see [40]) and on the original training set (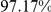), our approach results in a better performance for few labeled inputs. E.g., if 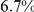 of the labels are used, we obtained 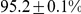 while the k-NN approaches achieved 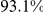. For still fewer labels the performance difference gets still more pronounced. On 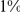 of the labels, the k-NN algorithm achieved just 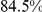 while our approach resulted in 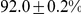 correct classifications. These results show a clear benefit of learning an unsupervised representation as provided by our approach, while fully supervised approaches such as k-NN algorithms can not make use of unlabeled data.

#### Unconstrained learning and unconstrained inputs

In the case of unconstrained EM, we use the original MNIST data (globally rescaled by a factor 1/255 to avoid numerical problems), with no input normalization. For the neural network results, artificial data is generated using the same blocks model as before, but without individually normalizing the generative fields. In the absence of input normalization, the contrast of the images remains unspecified; multiplying all inputs by an arbitrary constant does not affect the original model but can have serious consequences for learning on unconstrained data (intuitively, this scaling factor translates into an arbitrary change in learning rate, which is bound to affect learning). Hence, to facilitate the comparison between different models we globally rescale the generating fields to have the same mean intensity (averaged over all fields), while allowing different inputs to have different mean intensities (see Fig. 5A,B), using A = 200. Since the overall mean is preserved, any difference between the normalized and un-normalized data is not due to some overall scaling, but rather to constraining the space spanned by the data.

Learning with either constrained or unconstrained EM or the (linear) neural network proceeds as before, the difference being that either the normalized or the unnormalized data is used as input (learning rate 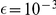 as before). Additionally, we use a variation of the linear neural circuit in which synapses change by simple Hebbian learning, followed by an explicit weight normalization, 
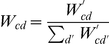
. This version ensures that the synaptic weights are still normalized to the constant 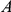 when the inputs are unconstrained. We use again 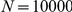 data points for training in all cases.

## Supporting Information

Text S1 Evolution of weights – details of derivations and approximations.
(PDF)
