# Supplementary material for: Feedforward Inhibition and Synaptic Scaling – Two Sides of the Same Coin?
Source: PLoS Comput Biol. 2012 Mar 22;8(3):e1002432. doi: 10.1371/journal.pcbi.1002432 (PMC3310709; doi:10.1371/journal.pcbi.1002432)
Supplement: Text S1 — Evolution of weights – details of derivations and approximations. (PDF) [file pcbi.1002432.s001.pdf]

Feedforward inhibition and synaptic scaling – two sides of the same coin?

Christian Keck[1,†], **Cristina Savin**[2,†], **Jörg Lücke**[1,3,*]

[1]Frankfurt Institute for Advanced Studies, Frankfurt am Main, Germany
[2]Computational and Biological Learning Lab, Department of Engineering, University of Cambridge, Cambridge, UK
[3]Department of Physics, Goethe-University, Frankfurt am Main, Germany
† These authors contributed equally to this work.
*E-mail: luecke@fias.uni-frankfurt.de

Text S1

The main intermediate steps of the derivation of the fixed point (main text, Eq. 5) were given in the Methods. Here, we discuss the details of the derivation.

Derivation of Equation 14 . First, we show that we obtain Eq. 14 (see Methods) if we apply Eq. 13 iteratively N times. For the derivation of Eq. 14, we will use two identities. First, note that for any sequence $R^{(n)}$ and $S^{(n)}$ the following holds:

$$\sum_{n=1}^{N} R^{(N-n)} \prod_{n'=n+1}^{N} (1 + S^{(N-n')}) + R^{(N)} \prod_{n'=1}^{N} (1 + S^{(N-n')}) \; = \; \sum_{n=1}^{N+1} R^{(N+1-n)} \prod_{n'=n+1}^{N+1} (1 + S^{(N+1-n')}) \, . \quad (1)$$

Second, note that for any sequence $S^{(n)}$ we have:

$$1 + \sum_{n=1}^{N} S^{(N-n)} \prod_{n'=n+1}^{N} (1 + S^{(N-n')}) \; = \; \prod_{n=1}^{N} (1 + S^{(N-n)}), \quad (2)$$

Eq. 1 is given in a straight-forward way by splitting the right-hand-side into a sum to N plus an additional term, and by changing the indices. Eq. 2 can be proven by induction and by making use of Eq. 1 for $R^{(n)} = S^{(n)}$. For both identities and in the following, we use the convention that for $n = N$ the product $\prod_{n'=n+1}^{N} X_{n'}$ is equal to one.

We will now show by induction that Eq. 14 holds. For $N = 1$ Eq. 14 is equal to Eq. 13 which verifies the base case. For the induction step, we start with Eq. 13 for $N + 1$ and insert Eq. 14 for $W_{cd}^{(T+N)}$, which

results in:

$$W_{cd}^{(T+N+1)} = A \frac{W_{cd}^{(T)} + \epsilon \sum_{n=1}^{N} F_{cd}^{(T+N-n)} \prod_{n'=n+1}^{N} (1 + \frac{\epsilon}{A} \sum_{d''} F_{cd''}^{(T+N-n')}) + \epsilon F_{cd}^{(T+N)} \prod_{n'=1}^{N} (1 + \frac{\epsilon}{A} \sum_{d''} F_{cd''}^{(T+N-n')})}{\sum_{d'} \big(W_{cd'}^{(T)} + \epsilon \sum_{n=1}^{N} F_{cd'}^{(T+N-n)} \prod_{n'=n+1}^{N} (1 + \frac{\epsilon}{A} \sum_{d''} F_{cd''}^{(T+N-n')}) + \epsilon F_{cd'}^{(T+N)} \prod_{n'=1}^{N} (1 + \frac{\epsilon}{A} \sum_{d'} F_{cd''}^{(T+N-n')}) \big)}$$

$$= \frac{W_{cd}^{(T)} + \epsilon \sum_{n=1}^{N+1} F_{cd}^{(T+N+1-n)} \prod_{n'=n+1}^{N+1} (1 + \frac{\epsilon}{A} \sum_{d''} F_{cd''}^{(T+N+1-n')})}{1 + \sum_{n=1}^{N+1} (\frac{\epsilon}{A} \sum_{d'} F_{cd'}^{(T+N+1-n)}) \prod_{n'=n+1}^{N+1} (1 + \frac{\epsilon}{A} \sum_{d''} F_{cd''}^{(T+N+1-n')})} \tag{3}$$

$$= \frac{W_{cd}^{(T)} + \epsilon \sum_{n=1}^{N+1} F_{cd}^{(T+N+1-n)} \prod_{n'=n+1}^{N+1} (1 + \frac{\epsilon}{A} \sum_{d''} F_{cd''}^{(T+N+1-n')})}{\prod_{n'=1}^{N+1} (1 + \frac{\epsilon}{A} \sum_{d'} F_{cd'}^{(T+N+1-n')})} . \tag{4}$$

To obtain (3) we applied identity (1) with $R^{(n')} = F_{cd}^{(T+n')}$ and $S^{(n')} = \frac{\epsilon}{A} \sum_{d'} F_{cd'}^{(T+n')}$. To obtain (4) we applied identity (2) with $S^{(n')} = \frac{\epsilon}{A} \sum_{d'} F_{cd'}^{(T+n')}$. The final expression (4) is identical to Eq. 14 for $N+1$, which completes the induction step and proves the claim.

Approximation 1. First, consider the product in the numerator of (14). If we rewrite the expression using $x = \exp(\log(x))$, we can apply a Taylor expansion for $\log(1+x)$ around $x = 0$. By keeping the linear term for small ϵ we obtain:

$$\prod_{n'=n+1}^{N} (1 + \frac{\epsilon}{A} \sum_{d'} F_{cd'}^{(T+N-n')}) = \exp\big(\sum_{n'=n+1}^{N} \log(1 + \frac{\epsilon}{A} \sum_{d'} F_{cd'}^{(T+N-n')}) \big)$$

$$\approx \exp\big(\frac{\epsilon}{A} \sum_{d'} \sum_{n'=n+1}^{N} F_{cd'}^{(T+N-n')} \big) \approx \exp\big(\frac{\epsilon}{A} (N-n) \sum_{d'} \hat{F}_{cd'}^{(n)} \big) , \tag{5}$$

By applying the approximation (5) to the numerator of (14) and for $n = 0$ to the denominator, we obtain (15).

Approximation 2.
Consider the sum over n in (15). For the summands with relatively small n, $\sum_{d'} \hat{F}_{cd'}^{(n)}$ is well approximated by the mean over N iterations, $\sum_d \hat{F}_{cd}^{(n)} \approx \sum_d \frac{1}{N} \sum_{n=1}^{N} F_{cd}^{(T+n)} = \hat{F}_c^{(0)}$. Only for n close to N we can expect the approximation to become inaccurate. Note, however, that the sum in (15) is dominated by summands with small values of n. This is because for small ϵ and large N the exponential factors are very large for n significantly smaller than N compared to factors with n close to N. We can therefore approximate:

$$\sum_{n=1}^{N} \exp\big(\frac{\epsilon}{A} (N-n) \sum_{d'} \hat{F}_{cd'}^{(n)} \big) F_{cd}^{(T+N-n)} \approx \exp\big(\frac{\epsilon}{A} N \sum_{d'} \hat{F}_{cd'}^{(0)} \big) \sum_{n=1}^{N} \exp\big(-\frac{\epsilon}{A} n \sum_{d'} \hat{F}_{cd'}^{(0)} \big) F_{cd}^{(T+N-n)} \tag{6}$$

The sum on the right-hand-side of (6) we now split into K parts with $\tilde{N}$ summands such that $\exp\big(-\frac{\epsilon}{A} n \sum_{d'} \hat{F}_{cd'}^{(0)} \big)$ changes little across each partial sum (note that we assume constant W). We

can then approximate:

$$\sum_{n=1}^{N} \exp\big(-\frac{\epsilon}{A} n \sum_{d'} \hat{F}_{cd'}^{(0)}\big)\, F_{cd}^{(T+N-n)} \;=\; \sum_{k=0}^{K-1} \sum_{n=k\tilde{N}}^{(k+1)\tilde{N}} \exp\big(-\frac{\epsilon}{A} n \sum_{d'} \hat{F}_{cd'}^{(0)}\big)\, F_{cd}^{(T+N-n)}$$
$$\approx \sum_{k=0}^{K-1} \sum_{n=k\tilde{N}}^{(k+1)\tilde{N}} \exp\big(-\frac{\epsilon}{A} n \sum_{d'} \hat{F}_{cd'}^{(0)}\big)\, \hat{F}_{cd}^{(0)} \;=\; \hat{F}_{cd}^{(0)} \sum_{n=1}^{N} \exp\big(-\frac{\epsilon}{A} n \sum_{d'} \hat{F}_{cd'}^{(0)}\big) \qquad (7)$$

If we apply (6) and (7) to Eq. 15 , we obtain Eq. 16 .

Approximation 3.
To obtain the left-hand-side of (17), we first observe that the sum over n in (16) can be written as a geometric series:

$$\sum_{n=1}^{N} q^n \;=\; \frac{q - q^{N+1}}{1-q} \;\approx\; \frac{q}{1-q} \;\text{ with }\; q = \exp\big(-\frac{\epsilon}{A} \sum_{d'} \hat{F}_{cd'}^{(0)}\big)\,. \qquad (8)$$

The approximation holds for small but finite ϵ and large N: q is smaller than one for small ϵ, which implies that q^{N+1} approaches zero for large N. By applying (8) to (16) and by observing that the first term in (16) is negligible for large N, we obtain (17).
